# Different Features of Bilingualism in Relation to Executive Functioning

**DOI:** 10.3389/fpsyg.2019.00269

**Published:** 2019-02-11

**Authors:** Daniel Eriksson Sörman, Patrik Hansson, Jessica Körning Ljungberg

**Affiliations:** Department of Psychology, Umeå University, Umeå, Sweden

**Keywords:** bilingualism, cognitive control, executive functioning, inhibition, switching, linguistic distance, middle age, old age

## Abstract

The notion that the long-term practice of managing two languages is beneficial for the executive control system is an ongoing debate. Criticism have been raised that studies demonstrating a bilingual advantage often suffer from small sample sizes, and do not control for fluid intelligence as a possible confound. Taking those suggested factors into account, focusing on older bilingual age groups and investigating the potential effects of linguistic distances, this study aimed to improve the interpretations of the bilinguals’ advantages. Measures of inhibition (Flanker, Stroop, Simon task) and switching (Number-letter, Color-Shape, Local-global task) were collected in participants in the ages 50–75 years (*n* = 193). Despite a large study sample, results did not support any beneficial effects related to improve processing costs in executive functioning. Sub-analyses of the two different language groups (Swedish–Finnish / Swedish–English) intended to investigate the effect of linguistic distances did not change this outcome. Future studies exploring the potential long-term term effects of bilingualism would benefit from identifying tests of cognitive control with greater ecological validity and include other measures of cognitive functioning. Language learning interventions may also be a promising tool for future research.

## Introduction

As we live longer, there is an urgent need to identify what factors may be beneficial for cognitive functioning in old age. Several factors have been suggested, such as engagement in physical exercise (e.g., Erickson and Kramer, 2009) as well as mentally stimulating activities (for reviews see e.g., Fratiglioni et al., 2004; Stern and Munn, 2010; Fallahpour et al., 2016). Another factor argued to improve memory functions is the ability to speak two languages, with numerous studies highlighting that bilingualism can promote superior cognitive performance (see e.g., Bialystok et al., 2009, 2012). Beneficial effects have been found in different domains such as for instance episodic memory recall (e.g., Schroeder and Marian, 2012; Ljungberg et al., 2013), dual-tasking (e.g., Bialystok et al., 2006; Sörman et al., 2017), verbal fluency (e.g., Ljungberg et al., 2013), working memory (for review, see e.g., Grundy and Timmer, 2017), and executive functioning (for reviews, see e.g., Bialystok et al., 2009, 2012). Measures of executive functioning have predominantly been focusing on single processes such as inhibition (e.g., Bialystok et al., 2004; Costa et al., 2009; Pelham and Abrams, 2014) and task-switching (e.g., Prior and Gollan, 2011; Wiseheart et al., 2016), which constitute two crucial components of cognitive control.

The proposal is that managing two or more languages puts demands on the cognitive control system, especially when switching between languages and suppressing the language not currently in use (Kroll et al., 2008). This constant exercise of cognitive control is assumed to generate transfer effects into cognitive tasks that taxes the same cognitive control network. To support this, it is posited that favorable effects found among bilinguals are related to the frequency with which bilinguals use and switch between languages in everyday life (Costa et al., 2009). In addition it is important to note that areas such as prefrontal cortex, anterior cingulate cortex and caudate, are needed for executive functioning, working memory, dual-tasking, and episodic memory recall, are regions also frequently activated when mastering two languages (Bialystok et al., 2009). For example, increased white matter integrity and gray matter density in the frontal lobe areas are purported to be positively related to bilingualism (Abutalebi et al., 2012; Gold et al., 2013a; Ma et al., 2014; Olulade et al., 2016); therefore, offering a neurological explanation of the associations found between these tasks and bilingualism.

However, the proposal that bilingualism is beneficial for cognitive ability, and executive functioning in particular, is not without controversy. Several studies outline that positive findings may be due to confounders not controlled for, such as differences in socioeconomic status, intelligence, culture, and immigration status (see Lehtonen et al., 2018). In addition, it is argued that bilingual advantages are most often found in studies with small sample sizes lacking statistical power (Paap et al., 2015). In a recent meta-analysis by Lehtonen et al. (2018) 152 studies with a research focus on the bilingual advantages on executive functioning where analyzed. Beneficial effects of bilingualism were detected in inhibition, switching, and working memory whilst a small disadvantage was found for bilinguals in verbal fluency, and no differences with regard to attention or monitoring was noted. However, of greatest importance, the bilingual advantages were not retained following adjustment for publication bias.

Another important factor to consider relates to when in an individual’s lifespan the second language was acquired (L2). It is proposed that lifelong bilingualism may enhance working memory connectivity, and that this could be an underlying mechanism explaining bilingual advantages in executive functioning (Luk et al., 2011). In addition to this, it may be important to consider proficiency in L2, such that a greater proficiency in L2 may cause increased cognitive demands (i.e., inhibitory control) when using L1 than a weak L2 would cause. It should be noted though that L1 still generally cause more interference than a strong L2 due to its strength. Green and Abutalebi (2013) argue that high proficiency in both L1 and L2 is most likely related to control of cross-language interference, with the frequent risk of intrusion errors from either L1 or L2; and that depends on the different interactional contexts the bilingual is within.

The linguistic distance between languages (i.e., the dissimilarity between languages with regard to for instance semantics and phonology) is another factor to take into consideration (Wichmann et al., 2010). In attempts to master two languages from the same language family, and/or languages that have a large semantic and lexical overlap, greater demands on the cognitive control system may be induced in efforts to avoid interference (Gollan et al., 2011). For example, Swedish and English are rooted in the same language family (i.e., Germanic languages); the use of these two languages in combination may incur greater demands on the inhibitory control system in comparison with Swedish and Finnish. The Finnish language constitutes a part of the Uralic language family; therefore a larger linguist distance exists between Swedish and Finnish (see e.g., McMahon and McMahon, 2005). However, there is a paucity of research in this area; further, within the limited number of studies conducted on the topic of linguistic distance an opposite effect has been observed when comparing English–Cantonese bilinguals with English–German or an English–French speaking group (Bialystok et al., 2005; Wierzbicki, 2014). Further research is warranted with studies powered by larger sample sizes and more extensive investigation of languages separated by varying levels of linguistic distance.

It is also important to note that the bilingual advantage is more evident for tasks that contain non-verbal material (e.g., Bialystok et al., 2014) compared to tasks that include verbal material, which could be a consequence of a smaller vocabulary among bilinguals in the language they are tested in compared to monolinguals (e.g., Bialystok et al., 2010; Bialystok and Luk, 2012). This smaller vocabulary size, in combination with the competition between languages, may cause greater demands on bilinguals when they perform executive tasks in experimental settings with verbal material. In line with this reasoning, beneficial effects are instead often found in non-verbal versions of inhibition tasks such as the Flanker task (Costa et al., 2009; Pelham and Abrams, 2014), the Simon Task (Bialystok et al., 2004), as well as non-verbal switching in the Color-shape task (e.g., Prior and Gollan, 2011; Wiseheart et al., 2016).

The inhibitory control function has been studied extensively and proposed as an underlying mechanism of the bilingual advantage (e.g., Green, 1998); but the general theory behind the bilingual benefits in executive functioning is argued to be an executive control process (Hernández et al., 2010; Hilchey and Klein, 2011) and the focus is not so much on a single inhibitory process (see Bialystok et al., 2012 for a review). The speakers need to monitor and switch between languages while inhibiting irrelevant information has been put forward as the explanation to why bilinguals outperform monolinguals in executive functioning tasks.

Many studies from a wide range of memory research fields have shown that several cognitive constructs are heavily influenced by aging, such as episodic memory (e.g., Rönnlund et al., 2005), dual-tasking (e.g., Verhaeghen and Cerella, 2002), working memory (e.g., Park and Payer, 2006; Sander et al., 2012) and executive functioning (e.g., Zelazo et al., 2004) including inhibition (e.g., Treitz et al., 2007), switching (e.g., Salthouse and Miles, 2002), and the ability to handle interference (e.g., Connelly et al., 1991). Studies of bilingualism have reached similar conclusions. For example, a study by Bialystok et al. (2008) observed decreased performance related to age in several memory tasks; however, the results also revealed that the older age group in the study benefited to a greater extent from bilingualism than the younger group. In both the study by Bialystok et al. (2004) and Salvatierra and Rosselli (2010) similar conclusions were made; when improved performance was detected in older age groups who performed the Simon task. Collectively, both studies imply that older bilinguals may have improved cognitive control compared to their monolingual counterparts. Taken together, these findings may be of relevance for the increasing older population in the western societies.

Two previous studies performed in Umeå, Sweden, investigated the effects of bilingualism on cognitive functioning in aging. The first, conducted by Ljungberg et al. (2013), included participants between 35 and 70 years at baseline. The authors found that late bilinguals outperformed monolinguals both at baseline and over time (15 years) in both letter fluency and episodic memory recall, although no differences were found with regard to change (interaction between time and language group). In a more recent study (Sörman et al., 2017), beneficial effects were found for bilingualism on dual-tasking at baseline measurement. Dual-task costs increased over time for bilinguals however, dual-task costs were stable for monolinguals. These findings were possibly influenced by less use of L2 in the bilingual group after retirement, since participants were all native Swedes living in Sweden. It should be noted that in both of the abovementioned studies, the test occasions for baseline measurement were either between 1988 and 1990 or 1993 and 1995. Most likely, this was a time when the number of “pure” monolinguals were more common in the Swedish society than today. The increasing number of immigrants and a more bilingual younger generation have influenced the Swedish society since then (Statistics Sweden, 2018). In addition, the Swedish educational system has for a long time encouraged knowledge of languages other than Swedish, and thus, most people living in Sweden today have knowledge in at least some other language (most commonly English that is taught through the formal educational system) than Swedish. The increased influence of television and social media in recent years, among other things, should also be stressed. Thus, the number of “pure” monolinguals are plausibly not as common in Sweden today as in the late 80s or the early 90s, making a classification of groups into bilinguals or monolinguals more difficult.

However, these changes do not preclude the possibility to investigate the effects of bilingualism. Luk and Bialystok (2013), for instance, suggested that bilingualism should not be considered as a categorical variable, but rather from a multidimensional perspective taking individual variation and different features of the bilingual experience into account. Thus, it might be worth considering different features of bilingualism on a continuum, rather than bilingualism as a distinct and static factor. Such a perspective on bilingualism also opens the possibility to investigate linear relationships between different features of bilingualism in relation to cognitive functioning. Furthermore, such an approach, using within-group analyses, decreases the risk of confounding variables influencing the results, which might be the case with the bilingual vs. monolingual approach, in which unobserved group differences may influence the results. So far, few studies have investigated the effects of language switching using a within-group correlative analyses method (see e.g., Soveri et al., 2011; Jylkkä et al., 2017). Recently, it was suggested that such method might be advantageous for advancing bilingual research (Lehtonen et al., 2018).

As noted, the executive control system is age sensitive; however, it is still unclear whether bilingualism can promote executive functioning later in life. Thus, the present study sought to investigate if two different features of bilingualism (estimated bilingualism and L2 proficiency), treated as continuous variables, were related to cognitive control studying a sample of 50–75 year old participants. Most participants (91.3%) had learned L2 after the age of 6 and thus to be considered as late bilinguals (e.g., Ljungberg et al., 2013; Sörman et al., 2017). Based on existing theories outlining that bilingual advantages are related to the concept of executive control (Bialystok et al., 2012), this study included six executive tasks, all related to domains of cognitive control. The choice of tasks was based on the findings presented in previous research literature highlighting that both inhibition (Ridderinkhof et al., 2004) and switching are processes related to cognitive control (e.g., Braver et al., 2003).

Since it has been argued that many previous studies demonstrating positive effects of bilingualism on executive functioning suffer from small sample sizes (Paap, 2014), this study included a large sample size to be able to increase power to the analyses. A within-group analyses approach was selected to minimize the influence of confounding variables on the results, also taking into account that bilingualism can be considered as a continuous variable. In addition, to avoid the possible impact of linguistic distance on cognitive functioning, and/or effects of immigration background on executive functioning, we aimed to perform sub-analyses with participants using languages within the same language family (English–Swedish). Further analyses of participants using languages from different language families (Finnish–Swedish) were conducted to determine if a linear trend could be observed between language skills and executive functioning in any of the groups. In addition, we included important covariates (age and fluid intelligence) that could potentially explain differences in cognitive control. Confounding variables like measures of intelligence, or the impact of language distances on cognitive control (e.g., Gollan et al., 2011) have not always been controlled for or matched when measuring bilinguals with a large mix of spoken languages (e.g., Paap and Greenberg, 2013). Based on the mixture of earlier findings and the increased control for confounders in the present study, an open hypothesis approach was used.

## Materials and Methods

### Participants

In the present study, data emanates from a project called “*Successful aging – A study of how bilingualism and choice of occupation contribute to preserve attention and memory across the adult life span”* that is an ongoing longitudinal study in Umeå, Sweden. Participants were recruited through pensioner’s and language associations (Finnish and English) and by advertisements in local newspapers. In the recruitment participants had to be between 50 and 75 years, and the criteria’s for participating were that (1) Participants considered themselves to be bilinguals and used both Swedish and Finnish regularly, or (2) Participants considered themselves to be bilinguals and used both Swedish and English regularly, or (3) Participants had none or minor knowledge in any other language than Swedish. Participants were invited to participate over two test sessions, about 1 week apart, both focusing on assessment of cognitive functions.

Participants with complete data on the language variables of interest was 210. A few cases with missing data on covariates (*n* = 14), and with no cognitive data available (*n* = 3) had to be excluded. Thus, data for a total of 193 were included in the present study. Most participants (91.3%) had learned L2 (non-native language) after the age of 6 and thus to be considered as late bilinguals. The mean age of this sample was 65.4 (*SD* = 5.8) and included a total of 118 women and 75 men. Only 7 in the total sample reported absolutely no knowledge in any other language than Swedish and could thus be considered as “pure” monolinguals. In total, 60 participants reported at least some prior knowledge in both Swedish and Finnish, and 122 rated at least some knowledge in both Swedish and English. A few participants (*n* = 4) had another L2 than Swedish, English or Finnish (German = 3; Spanish = 1). Based on comprehensive data collected on medical history, the high accuracy levels on the cognitive tasks, and subjective evaluations by trained test leaders, all participants could be confirmed to be neurologically healthy. All participants had normal or corrected-to-normal vision during testing and reported correct color vision. The major part of participants were right-handed (94,8%).

Participant characteristics, for the total sample and as function of language combination, are provided in Table 1.

**Table 1:** Participant characteristics for the total sample and for each language combination are provided in this Table.

	Total sample (*n* = 193)				Swedish–Finnish (*n* = 60)				Swedish–English (*n* = 122)			
	Mean	*SD*	Range	%	Mean	*SD*	Range	%	Mean	*SD*	Range	%
Age	65.4	5.8	50–75		63.7	6.8	50–74		66.0	5.1	52–75	
Ravens (*Gf*)	5.1	2.7	0–11		5.1	3.0	0–11		5.2	2.4	0–11	
Estimated Bilingualism	5.2	3.5	0–10		8.5	1.4	4–10		3.8	3.0	0–10	
L2 Proficiency	6.1	2.8	0–10		8.1	1.5	3.5–10		5.4	2.5	0–10	
AoA – L2	11.7	4.9	0–27		12.2	6.4	0–27		11.4	3.8	0–27	
Swedish as L2	13.1	6.6	0–27	32.1	12.5	6.2	0–27	96.7	23.3	3.5	20–27	3.3
Non-Swedish as L2	10.9	3.4	0–23	64.2	4.5	6.3	0–9	3.3	11.1	3.2	0–23	96.7

*SD, standard deviation; Gf, general fluid ability; L2, second language; AoA, age of acquisition*.

Within the Swedish–Finnish group (*n* = 60), most were born outside Sweden (56 in Finland, 1 in Russia) and moved to Sweden at a mean age of 20.79 (*SD* = 8.68) years. Thus, most (96.7%) had Finnish as L1 (AoA = 0). It is worth to note that after living in Sweden for decades, some today referred to Swedish as their dominant language (*n* = 17). As can be seen, most participants had prior knowledge in Swedish before moving to Sweden (Swedish is an official language in Finland and a part of the educational system) since mean AoA for Swedish as L2 was lower (12.54) than the overall mean age moving to Sweden.

In the Swedish–English group, 118 (96.7%) were born in Sweden and had Swedish as L1 (AoA = 0), and 4 (3.3%) were born in another country with English as L1 (AoA = 0). Mean age for moving to Sweden among the foreign born was 22.83 (*SD* = 12.64). Many participants that had English as L2 started to learn it at school since English constitutes a part of the Swedish educational system and begins to be taught during primary school.

Participants on the Swedish–English continuum were somewhat older compared to the Swedish–Finnish group, although comparable *Gf*. However, groups also differed on Estimated Bilingualism and L2 Proficiency with the Finnish–Swedish group having higher scores.

### Measures

#### Bilingualism

Two different measures were used. (1) Estimated Bilingualism. All participants rated their level of bilingualism on a scale ranging from 0 (monolingual) to 10 (bilingual). Preliminary analyses indicates that this single-item question correlates well (*r* = 0.79) with the “Bilingual Score” calculated from a Swedish translation of a recent version of the Language and Social Background Questionnaire (LSBQ) developed by The Lifespan Cognition and Development Lab at the Department of Psychology, York University^1^. (2) Proficiency in L2. Similar to the procedure previously used (see Ljungberg et al., 2013; Sörman et al., 2017) participants rated their ability to speak, write, read, and listen in their L2. A mean value of these features was calculated ranging from 0 to 10.

#### Executive Functioning

All cognitive tasks were programmed in E-Prime 2.0 professional (Psychology Software Tools, Pittsburgh, PA). For each task, participants were instructed to respond as quickly and as accurate as possible. RTs were calculated only on correct responses. In addition, and to ensure correct implementation, an accuracy level of 75% or more was used for each task to be included for further analyses. Thus, the number of participants used in the analyses differed dependent on cognitive measure used as dependent variable.

#### Inhibition

The first computerized task to measure individual differences in inhibitory control was the Flanker task (Eriksen and Eriksen, 1974). After a fixation cross (+) had been displayed for 2000 ms, five arrows were presented at the center of the screen (e.g., < < > < <) and the participants had to decide whether the arrow at the center of the string was pointing to the left (press “X” key) or the right (press “M” key) while ignoring the direction of the flanker arrows. In the congruent condition, flanker arrows pointed in the same direction as the central target. In the incongruent condition, the flankers pointed in the opposite direction as the central target. After 10 practice trials, participants performed 96 (6^∗^16) test trials, of which half of the trials were congruent, and half incongruent. The stimuli remained for 2000 ms if no response was given. The Flanker effect, calculated as the difference in average response time between congruent and incongruent trials, was used as dependent measure in the analyses.

The second task used to tap inhibitory control was the Stroop task (Stroop, 1935; Lu and Proctor, 1995). This was the only task used in this study that included verbal information (words). After a fixation cross had been displayed for 600 ms, color words were presented to the participants and they were instructed to name the color of the ink in which the color word was written. For each word, participants were presented with two alternative answers, one on each side of the stimulus word. In congruent trials, the color of the ink matched the name of the color (e.g., red written in red ink). In incongruent trials, there color of the ink did not match the name of the color (e.g., red written in green ink). Participants pressed the “X” key for the alternative answer to the left of the stimulus word, and the “M” key for the alternative to the right. After 6 practice trials, participants performed 96 (2^∗^48) test trials. The stroop effect, that is, the difference in average response time between congruent and incongruent trials, was used as dependent measure.

The third task used to measure inhibitory control was the Simon task (Simon and Wolf, 1963) based on the version described by Bialystok et al. (2004). Each trial started with a fixation cross that was that was displayed for 800 ms followed by 250-ms blank interval. Then a green or red square appeared either on the left or the right side of the screen. Independent of position, participants were instructed to press the “M” key if the square was green, and press the “X” key if the square was red. In the congruent condition, the colored square were on the same side on the screen as the associated response key on the keyboard. In the incongruent trails, the colored square were on the opposite side as the associated response key. Participants had to respond to each to the stimuli within 1000 ms before the next trial started and each trail was separated by a 500 ms response-to-stimulus interval. After 20 practice trials, participants performed 2^∗^40 trials, half of which were incongruent trials and half of which were congruent trials. The difference in average response time between congruent and incongruent trials (i.e., the Simon effect) were used as dependent measure.

#### Switching

A modified version of the number-letter task (Rogers and Monsell, 1995) was the first task used to tap the switching function. In this task, a number–letter pair (e.g., 5A) appeared in one of the four quadrants on the computer screen. If the pair was presented in any of the top quadrants, participants were instructed to decide if the number was odd or even by pressing either the “X” key (odd) or the “M” key (even). Numbers 2, 4, 6, and 8 was used for even numbers, and 3, 5, 7, and 9 for odd numbers. If the pair was presented in any of the bottom quadrants, they were told to respond to whether the letter was a lower case (“X” key) or upper case (“M” key) letter. Letters A, E, I, and U were used for upper case letters, and g, k, m, and r for lower case letters. In alternate versions of the task, participants are instructed to respond to whether the letter is a consonant or vowel. But since the regular use of these speech sounds differ between languages (e.g., between Finnish and Swedish), the consonant-vowel categorization was not used in the present study. All blocks were preceded by 8 practice trials. In the first block, participants performed test 32 trials with the pair presented in any of the top quadrants (i.e., categorize numbers). Similarly, in the second block, participants performed 32 trials with the pair in any of the bottom quadrants (i.e., categorize letters). Finally, in the third mixed trial block, including 128 trials, pairs rotated clockwise around all four quadrants. Half of the trials required a mental shift between categorization number to letters or vice versa. Similar to Miyake et al. (2000) the switch cost was calculated as the difference between the average RTs of the trials were mental shift was required, and the average RTs of the trials from the blocks were no shift was required.

The second task used to tap the switching function was the Color-Shape Task. The task was similar to versions previously used (see Prior and MacWhinney, 2010; Paap and Greenberg, 2013). In all blocks and trials, the stimuli was either a red circle, a red triangle, a blue circle or a blue triangle. Each trial always began with a center fixation cross shown for 350 ms followed by a blank screen for 150 ms before the symbol was presented. Two dummy trials always started each block. In the first block, participant’s task was to only identify the color of symbols (circles and triangles). If the symbol was blue, participants responded by pressing the “Z” key with their left middle finger. If the symbol was red, participants responded by pressing the “X” key with their left index finger. In the second block, participants only had to make shape decisions. Participants pressed the “M” key with their right middle finger if the symbol was a triangle, and the “N” key with their left index finger if it was a circle. Each of the first two blocks started with 8 practice trials followed by 36 test trails. In the final mixed-task blocks, a pre-cue was shown for 250 ms prior to symbol and remained above the symbol until a response was made. If the pre-cue was a rainbow, participants were instructed to decide the color of the symbol. If the pre-cue was a black circle embedded within a black triangle, participants had to make shape decisions. The mixed-task blocks started with 16 practice trials followed by 3^∗^48 test trials, with equal number of repeat and switch trials. Switching costs were calculated as the difference in average RTs on switch trials as opposed to non-switch/repeat trials in the third block. Mixing costs (monitoring) was calculated as the difference between average RT in the single-task blocks and average RT on non-switch trials in the third mixed-task blocks.

The third task used as indicator of switching ability, the Local-Global task (Navon, 1977), was similar to the one used by Miyake et al. (2000). In all blocks and trials, a geometric figure was presented at the center of the screen and was either a circle, square, triangle or cross. Each of these ”Global” figures also composed of smaller so called ”local” figures that could be any of the same geometric figures that were used for the global symbols, i.e., a global ”Circle” could be composed by smaller “Squares.” The task was to decide the form on either the “Global Figure” or the “Local Figures” in each trial dependent on the color of the figure (blue = global; black = local). A fixation cross was displayed for 350 ms and then the symbol was presented. Participants pressed the “1” key for a circle (i.e., 1 line), “2” for a cross (2 lines), “3” for a triangle (3 lines), and “4” for a square. Each trail was separated by a 500 ms response-to-stimulus interval. The test started with 38 practice trials followed by 98 test trials. Half of the trials required switch from local to global (or vice versa) and half required no mental switch. The switch cost was calculated as the difference in average RTs between switch trials and non-switch trials.

#### Covariates

Age and fluid intelligence (*Gf*) were included as covariates in the analyses. A 12-item short form of The Raven Advanced Progressive Matrices Test (Arthur and Day, 1994) was used as indicator of *Gf*. This test has essentially the same measuring properties as the original form (*r* = 0.90).

### Statistical Analysis

Within-group analyses were used in which each aspect of bilingualism was considered as a continuous variable. In the sub-analyses of language groups, with the purpose to investigate the influence of linguistic distance, we also performed within groups analyses. The reason to avoid comparisons of language groups was to reduce the influence of confounding variables such as foreign background, cultural differences, and the context were groups learned L2 influencing the results. Also, since it was found that language groups differed on the mean level on aspects of bilinguals relevant to this study, further support was given to analyze language groups separately.

A two-step hierarchical multiple regression analyses were used, with RT processing cost used as dependent variable. In step 1 (i.e., model 1) of all analyses, we included age and *Gf* as independent variables (*Y =* β_0_ + β_1_X_1_ + β_2_X_2_ + *e*; *Y* = dependent variable, β_0_ = intercept, X_1_ – X_2_ = independent variables, β_1_ – β_2_ = estimated regression coefficients, *e* = error residual). In Step 2 (i.e., model 2), features of bilingualism were entered (each in separate models) as predictors of performance on each executive task (*Y =* β_0_ + β_1_X_1_ + β_2_X_2_ + β_3_X_3_ + *e;* X_3_ = bilingualism variable). The two-step approach were used to be able to calculate R square Change (*ΔR^2^*), that is, the unique explained variance of each bilingualism variable entered to the model. Bayesian factors (BF) were also included in order to help interpret findings. BF is the ratio of the likelihood of one particular hypothesis (H_1_) to the likelihood of another (H_0_). Due to low correlations between executive tasks, *r* range 0.03–0.10 for the inhibition tasks, and *r* range 0.15–0.22 with regard to the switching tasks, and low Cronbach’s α for the three inhibitory (0.08) and switching (0.25) tasks, we did not compute composites scores of each construct which could have been valuable with regard to the research question/s. All data were analyzed with SPSS version 25 (IBM Corp, 2017).

## Results

Descriptive data of processing cost in RTs together with mean values for conditions (congruent – incongruent / switching – non-switching) of each sample and in each executive task are presented in Table 2.

**Table 2:** Performance on each executive task.

	Total sample		Swedish–Finnish		Swedish–English	
	RT in ms		RT in ms		RT in ms	
Task	Mean	*SD*	Mean	*SD*	Mean	*SD*
**Flanker task**						
Congruent	635.8	99.9	641.3	123.5	635.1	88.6
Incongruent	734.4	116.3	736.6	142.2	735.9	103.9
Flanker effect	98.6	56.3	95.2	60.5	100.8	55.8
	*n* = 172		*n* = 52		*n* = 112	
**Stroop task**						
Congruent	1591.7	437.7	1652.1	549.7	1566.2	394.3
Incongruent	1845.8	509.9	1941.9	642.7	1805.1	453.1
Stroop effect	254.1	173.8	289.8	170.7	238.9	175.2
	*n* = 159		*n* = 44		*n* = 109	
**Simon task**						
Congruent	547.6	80.0	553.4	82.1	544.5	77.6
Incongruent	584.6	75.3	590.9	86.0	580.9	69.5
Simon effect	36.9	34.4	37.5	33.8	36.3	35.1
	*n* = 184		*n* = 57		*n* = 117	
**Number letter**						
Non-Switch trials	1443.4	443.2	1432.2	428.5	1421.4	396.3
Switch trials	2323.4	824.2	2304.5	798.8	2314.8	817.3
Switch cost	880.0	639.2	872.3	648.5	893.5	643.7
	*n* = 176		*n* = 52		*n* = 114	
**Color Shape**						
Single-task trials	679.5	165.2	696.7	177.2	669.2	160.8
Non-Switch trials	1142.0	252.5	1134.4	285.0	1135.9	230.7
Switch trials	1310.7	289.7	1319.7	312.1	1296.8	270.1
Mixing cost	477.3	215.7	454.9	205.0	481.4	211.5
(monitoring)
Switch cost	168.6	133.0	185.4	144.5	160.9	127.1
	*n* = 169		*n* = 49		*n* = 113	
**Local global**						
Non-Switch trials	2629.0	1122.4	2721.5	1544.6	2569.1	872.3
Switch trials	2871.0	1173.1	2943.3	1574.6	2817.3	932.3
Switch cost	242.0	311.9	221.8	251.8	248.2	329.5
	*n* = 178		*n* = 53		*n* = 116	

*SD, standard deviation; RT, reaction time; ms, milliseconds*.

For all RT data, analyses of both skewness (range -0.07 to 0.92) and kurtosis (range -0.28 to 1.03) revealed normally distributed data. Suggested thresholds in the literature is 2 for skewness, and 7 for kurtosis (Finney and DiStefano, 2006). Also all independent variables (age, *Gf*, estimated bilingualism, L2 proficiency) used in the analyses were normality distributed with regard to skewness (range -0.65 to 0.39) and kurtosis (-1.40 to 0.35).

Next, hierarchical multiple regression analyses were executed. The results are presented in Tables 3, 4.

**Table 3:** Results from the hierarchical regression analyses with the bilingualism variables as predictors of processing cost in the inhibition tasks.

			Flanker Effect					Stroop Effect					Simon Effect				
			*ΔR^2^*	R	β	*p*	*BF^∗^*	*ΔR^2^*	R	β	*p*	*BF^∗^*	*ΔR^2^*	R	β	*p*	*BF^∗^*
**Total sample**	Step 1^a^	(Covariates)	0.021			0.16		0.143			<**0.001**		0.039			**0.03**	
	Step 2^b^	Estimated Bilingualism	0.002	0.023	-0.042	0.59	0.095	0.020	0.163	0.145	0.06	0.073	0.000	0.039	-0.015	0.84	0.081
		L2 Proficiency	0.000	0.021	0.020	0.80	0.061	0.018	0.161	0.135	0.07	0.079	0.000	0.039	-0.012	0.87	0.076
**Swedish–Finnish**	Step 1^a^	(Covariates)	0.048			0.30		0.218			**0**.**01**		0.048			0.26	
	Step 2^b^	Estimated Bilingualism	0.001	0.049	-0.034	0.83	0.109	0.005	0.222	-0.074	0.63	0.348	0.002	0.050	-0.049	0.75	0.154
		L2 Proficiency	0.012	0.060	0.114	0.44	0.189	0.017	0.235	0.139	0.35	0.122	0.039	0.088	0.208	0.14	0.189
**Swedish–English**	Step 1^a^	(Covariates)	0.053			0.05		0.146			<**0**.**001**		0.058			**0.03**	
	Step 2^b^	Estimated Bilingualism	0.000	0.053	-0.023	0.81	0.102	0.002	0.148	0.050	0.59	0.077	0.001	0.059	-0.033	0.73	0.119
		L2 Proficiency	0.001	0.054	0.025	0.79	0.080	0.004	0.150	0.061	0.51	0.076	0.005	0.063	-0.073	0.44	0.173

*^*a*^Step 1 (Model 1), Adjusted for age and fluid intelligence; ^*b*^Step 2 (Model 2), Measures of bilingualism were included, and investigated in separate models; L2, Second Language*; ΔR^*2*^, R square Change*; R, Total R square; β, Standardized Beta; *BF*, Bayes factor; ^∗^Without covariates. Bold values denote statistical significance*.

**Table 4:** Results from the hierarchical regression analyses with the bilingualism variables as predictors of processing cost in the switching tasks.

													Color shape – Mixing cost									
			Number letter – Switch cost					Color shape – Switch cost					(monitoring)					Local global – Switch cost				
			*ΔR^2^*	*R*	β	*p*	*BF^∗^*	*ΔR^2^*	*R*	β	*p*	*BF^∗^*	*ΔR^2^*	*R*	β	*p*	*BF^∗^*	*ΔR^2^*	*R*	β	*p*	*BF^∗^*
**Total**	Step 1^a^	(Covariates)	0.042			**0**.**024**		0.018			0.23		0.144			<**0**.**001**		0.012			0.33	
**sample**	Step 2^b^	Estimated Bilingualism	0.002	0.044	0.044	0.59	0.061	0.001	0.018	0.030	0.72	0.061	0.002	0.146	0.042	0.58	0.092	0.001	0.013	0.027	0.72	0.059
		L2 Proficiency	0.001	0.043	-0.025	0.74	0.093	0.001	0.019	0.032	0.68	0.062	0.001	0.145	-0.025	0.74	0.172	0.000	0.012	0.000	0.99	0.062
**Swedish–**	Step 1^a^	(Covariates)	0.089			0.10		0.006			0.86		0.324			<**0**.**001**		0.065			0.19	
**Finnish**	Step 2^b^	Estimated Bilingualism	0.016	0.104	0.135	0.37	0.177	0.000	0.006	-0.017	0.92	0.116	0.004	0.328	-0.069	0.60	0.174	0.006	0.071	0.086	0.57	0.164
		L2 Proficiency	0.008	0.096	0.090	0.53	0.173	0.047	0.053	0.222	0.14	0.297	0.004	0.328	-0.063	0.62	0.112	0.053	0.118	0.241	0.09	0.900
**Swedish–**	Step 1^a^	(Covariates)	0.116			**0**.**001**		0.033			0.16		0.86			**0**.**01**		0.028			0.21	
**English**	Step 2^b^	Estimated Bilingualism	0.003	0.119	0.060	0.52	0.083	0.000	0.034	-0.020	0.84	0.094	0.000	0.086	-0.004	0.97	0.089	0.003	0.031	0.053	0.58	0.074
		L2 Proficiency	0.005	0.121	-0.072	0.44	0.253	0.000	0.034	-0.017	0.86	0.088	0.011	0.097	-0.108	0.25	0.289	0.000	0.028	-0.022	0.82	0.088

*^*a*^Step 1 (Model 1), adjusted for age and fluid intelligence; ^*b*^Step 2 (Model 2), measures of bilingualism were included, and investigated in separate models; L2, second language*; ΔR^*2*^, R* square change; R, total R square; β, standardized beta; *BF*, bayes factor; ^∗^Without covariates. Bold values denote statistical significance*.

Hierarchical multiple regression models of the total sample revealed that step 1, which included covariates age, and *Gf*, resulted in significant increment in the explained variance for the Stroop effect, Δ*R^2^* = 0.143, *F*(2,156) = 13.03, *p* < 0.001, the Simon effect, Δ*R^2^* = 0.039, *F*(2,181) = 3.64, *p* = 0.028, Number-Letter switch cost, Δ*R^2^* = 0.042, *F*(2,173) = 3.82, *p* = 0.024, and Color-Shape Mixing Cost, Δ*R^2^* = 0.144, *F*(2,166) = 13.96, *p* < 0.001. Age had its largest effect on Color-Shape mixing cost (β = 0.29, *p* < 0.001) and the Stroop effect (β = 0.26, *p* < 0.001). *Gf* had significant influence on Color-Shape mixing cost (β = -0.179, *p* = 0.05) and the Stroop effect (β = -0.179, *p* < 0.04), but were also borderline significant for the Simon effect (β = -0.179, *p* = 0.053), Number-letter switch cost (β = -0.157, *p* = 0.06), and Color-Shape switch cost (-0.146, *p* = 0.09). With regard to bilingualism, no predictive effect were found for any of these variables. For the Stroop task, bilingual variables almost reached statistical significance (Estimated bilingualism β = 0.145, *p* = 0.06; L2 Proficiency β = 0.135, *p* = 0.07), although indicative of a bilingual disadvantage. Analyses of the subsamples, i.e., the Swedish–English and Swedish–Finnish language groups, revealed similar results as in the analyses of the total sample.

As can be seen in Tables 3, 4, Bayes factors revealed that there were substantial to strong evidence in favor of null hypothesis in most models that included bilingualism variables (entered separately) as predictors of performance (BF range = 0.059–0.297). A BF within range 0.03–0.10 is suggested to be in strong support of H_0_, and a BF within 0.10–0.33 is considered to be in substantial favor of H_0_, In the Swedish–Finnish group, there was anecdotal evidence (BF = 0.90, within the anecdotal range of 0.33–1.00) in favor of the null hypothesis in the model that included L2 proficiency as predictor of performance in the Local-Global task, as well as anecdotal evidence (BF = 0.35) in favor of the null hypothesis in the model that included estimated bilingualism as predictor of performance in the Stroop task. Overall, results from Bayesian analyses are in favor of H_0_, suggesting that bilingualism is not related to performance in any of the tasks.

### Additional Analyses

We conducted a series of *post hoc* additional analyses to rule out the possibility of alternative associations between bilingualism (Estimated bilingualism and L2 proficiency) and executive functioning other than those defined at the beginning of this study. Similar to the main analyses, hierarchical multiple regression models were used, with age and *Gf* included as covariates in the first step. Although the sample mostly included late bilinguals (91.3%) that learned L2 after the age of 6 years, analyses with age of acquisition (AoA) were executed. Results from these analyses revealed no associations between AoA and processing cost, in any of the tasks. Neither did additional analyses show that bilingualism was related to global RT or accuracy levels in any condition (overall, congruent, incongruent, switching, non-switching) in any task.

Analyses were also performed using the extremes on the bilingual scale, that is, comparing those with the highest score (9–10) on the English–Swedish and the Finnish–Swedish groups with those having the absolute lowest score (0–1). Results did not show that those on the highest end of the continuum had any advantages compared to those on the lowest end on any of the tasks (RTs and Accuracy variables). Finally, analyses without *Gf* as covariate did not the change the overall findings in this study.

## Discussion

The objective of this study was to investigate whether bilingualism could predict performance in executive functioning in a sample aged between 50 and 75 years at the time of measurement. In the analyses we included two indicators of bilingualism: estimated bilingualism, and L2 proficiency. The study also aimed to investigate possible cognitive variations due to language distances (e.g., Gollan et al., 2011). We adopted a within-group analysis approach and adjusted for covariates (age and *Gf*) that could potentially influence differences on executive functioning. Models showed no favorable effects of bilingualism on cognitive control. Sub-group analyses of participants switching between languages within the same language family (English–Swedish), and analyses of participants switching between different language families (Finnish–Swedish) did not change this outcome. Thus, results do not support that linguistic distance had an impact on the effects on the cognitive control system measured with performances in inhibition and switching tasks. The overall findings were also supported by results from Bayesian statistics and the likelihood that Bilingualism is not related to performance in executive functioning.

The present study were not able to detect any differences due to levels of bilingualism and a possible increase in executive functioning in older age groups. As noted, the executive control system often declines with age, and it is of critical importance to identify factors that promote the executive control system. Previously, numerous studies have suggested that bilingualism might be such factor, demonstrating beneficial effects of bilingualism on executive functioning (for reviews, see e.g., Bialystok et al., 2009, 2012) despite some studies arguing that executive functions are almost entirely driven by genetics and one of the most heritable psychological traits (Friedman et al., 2008). Our data could not support any bilingual benefits on executive functions; instead findings are in line with those suggesting no favorable effects of bilingualism on the executive control system (see e.g., Lehtonen et al., 2018). In addition to this, we had two sub-samples that differed with regard to language combination and background, but neither of the sub-group analyses of these groups changed the outcome.

The results of the present study are at odds with previous research findings on older age groups (e.g., Gold et al., 2013b; Bialystok et al., 2014), but also in accordance with other studies that have not been able to demonstrate any bilingual advantages (e.g., Kirk et al., 2014). In a study by Kousaie et al. (2014), for instance, comparing French/English bilinguals (*M* = 70.69 years) with monolingual anglophones and monolingual francophones, using several executive function tasks, including the Stroop task, the Simon task, Digit span, Sustained attention to response task, and the Wisconsin Card Sort Test, no clear evidence of a bilingual advantage was found. However, English and French are not a part of the same branch within the Indo-European language family, since French constitutes a part of the Romance branch, whereas, as previously noted, English constitutes a part of the Germanic languages of the Indo-European language family (e.g., McMahon and McMahon, 2005). Thus they did not include bilinguals using languages characterized by near linguistic distances. In our study, we included two different language combinations to be able to investigate whether linguistic distance, and possible increased or decreased cognitive demands thereof, would influence executive functions differently. However, after separate within group analyses (near distance group = Swedish–English, far distance group = Swedish–Finnish), we did not find any signs of a linear relationships between higher values on features of bilingualism and performance on the executive tasks. Important to note is that these findings were not adjusted for multiple comparisons, an adjustment that would have made it even more difficult to detect significant effects of bilingualism. Therefore, results from our study confirms but also builds upon the findings of Kousaie et al. (2014).

This was not the first study to include bilinguals that use Swedish and Finnish in their everyday life. Soveri et al. (2011), for instance, found limited relationships between balanced language use in everyday life and performance in executive tasks in a sample of 30–75 year old simultaneous Finnish–Swedish bilinguals. They found, however, beneficial effects of bilingualism on their measure of language switching. In our study, most participants (91.3%) had learned L2 after the age of 6, and thus are considered as late bilinguals. It has been suggested that the earlier AoA of L2, the larger the bilingual advantage (Luk et al., 2011). Learning two languages in young ages may not only rely on different neural and cognitive systems, it may as well involve earlier development of neural systems. In addition to this, learning in early in life is often much more embodied. Hence, it has been speculated about the possibility that it is rather AoA than the constant use of two languages that leads to a beneficial effects on cognitive functioning in older adults (see e.g., Hernandez et al., 2018). Although it is plausible that our results would have differed with a sample that only included early simultaneous Finnish–Swedish bilinguals; it should also be noted that some studies argue that beneficial effects of bilingualism should be present also when L2 is learned later in life (Ljungberg et al., 2013; Sörman et al., 2017). Although our sample, included mostly late bilinguals, a comparison of AoA for the few early bilinguals with the late bilinguals did not change the overall outcome of this study.

One interesting aspect of this study is that one of the tasks used (i.e., Stroop task), includes lexical information. It has been shown that the bilingual advantage more often is evident for tasks that contain non-verbal material (Bialystok et al., 2014) and a recent study measuring young adults showed bilingual effects on an ambiguous figure task measuring selective attention (Chung-Fat-Yim et al., 2017). Although we could not find any significant bilingual advantages in any of the non-verbal tasks, in the Stroop task however, a descriptive non-significant opposite pattern was detected. The relationship was in a direction of a bilingual disadvantage. It is therefore plausible that with an even larger sample size we would have been able to confirm that bilinguals have a disadvantage in executive tasks that contain verbal information.

The difference in mean RTs between conditions (congruent – incongruent / switching – non-switching) is commonly used as measures of conflict processing for both inhibitory control or switching ability. However, our data showed that correlations were extremely low between the inhibition tasks, but also low for the switching tasks; thus, indicating poor task validity. This is not a new phenomenon though, and previous studies have reported problems with low correlations between executive tasks (see e.g., Paap and Sawi, 2014). Although these issues are not restricted to bilingual research, low validity stresses whether these tasks are trustworthy tools to investigate the suggested bilingual advantage. To some extent, these issues with the measures might help to explain why results are scattered on all sides in regards to the advantages and disadvantages of bilingualism. Considering that longitudinal beneficial effects of bilingualism have been obtained in our own lab in other cognitive tasks such as episodic recall, verbal fluency and dual-tasking (Ljungberg et al., 2013; Sörman et al., 2017) differences in task validity and reliability between previous studies and this study might have to be taken into account. Paap et al. (2014, p. 618) states that, “a compelling demonstration of a bilingual advantage should show significant advantages on the same component of executive functioning in two different tasks thus demonstrating convergent validity.” As it seems, it may be difficult to be compelling considering the validity of the executive tasks. With regard to episodic recall, for instance, that were used by Ljungberg et al. (2013), 5- and 10-year stability coefficients of 0.83 and 0.82 have been reported. Test-retest values for some of measures used in the present study overall have been reported to be lower (Flanker effect = 0.52; Simon effect = 0.43; Color-shape switch cost = 61; Color-shape mixing cost 0.75, see Paap and Oliver, 2016). Although the reliability of the processing cost measures must be stressed it is important to put forward that additional analyses, including other measures from the tasks, such as accuracy scores and global RTs, did not change the patterns found in the main analyses.

This investigation of the relationship between bilingualism and executive functioning is to our knowledge one of the first that combine the aging perspective, linguistic distance, various measures of inhibition and task-switching, using a larger sample size, within a single study design. Since it has also been suggested that differences in executive functioning can be linked to cultural differences (Morton, 2010), we minimized the influence of cultural factors using a within groups analyses approach and investigated the language groups separately. Thus, this study hopefully adds another piece to the puzzle and provides new knowledge to the research field. The results builds on previous cross-sectional results that indicate bilingualism is not related to executive functioning (for a recent meta-analyses, see Lehtonen et al., 2018). Another strength of this study was that we included *Gf* as covariate, which was deemed necessary based on noted shortcomings in previous studies.

Despite the strengths of this study, some limitations are to be acknowledged. Although the aim was to investigate two different language groups separately, based on factors previously mentioned, it was found that the bilingual groups were not equal to some extent. The Swedish–Finnish group rated higher values and showed lower standard deviation on both estimated bilingualism and L2 proficiency compared to the Swedish–English group. These differences may have concealed effects related to linguistic distance. For instance, that many participants amongst the Finnish speakers were on the higher end of the continuum may have masked trends that could have been observed with a somewhat larger spread in the data. Although additional analyses comparing the extremes on both language continuums did not change the outcome, we cannot totally rule out the possibility. Also, since bilingualism have been found to be related to superior performance in domains such as episodic memory recall (e.g., Schroeder and Marian, 2012; Ljungberg et al., 2013), dual-tasking (e.g., Bialystok et al., 2006; Sörman et al., 2017), and working memory (for review, see e.g., Grundy and Timmer, 2017), it is still possible that linguistic distance may influence other aspects of cognitive functioning than those included in this study.

It is also possible that more sensitive instruments to measure aspects of bilinguals may have altered the results. In this study, we used participants’ subjective estimation of bilingualism and expertise. It should be noted that in previous studies, beneficial effects of bilingualism using similar subjective measures of language skills have been reported (see Ljungberg et al., 2013; Sörman et al., 2017). As previously noted, preliminary analyses indicate the single item question of estimated bilingualism used in the present study correlated highly (*r* = 0.79) with the “Bilingual Score” calculated from a Swedish translation of a recent version of the Language and Social Background Questionnaire, supposed to measure level of bilingualism. Apart from our own studies, other studies have convincingly shown that self-reported measures tend to highly correlate with objective and standardized measures of language proficiency (e.g., Marian et al., 2007).

It must also be stressed that the sample used in the present study were almost without any so called “pure” monolinguals, that is, possessing no knowledge of any other language than Swedish. In prior studies (Ljungberg et al., 2013; Sörman et al., 2017), participants included and categorized as monolinguals were basically without any other language skills than Swedish. However, the present study was performed approximately 30 years after the baseline measurement were taken in previous studies, in the elapsed time the number of pure monolinguals has decreased and the number of bilinguals increased in the Swedish society. Thus, finding “pure” monolinguals appears to be a challenging task in Sweden today. Although many participants may have subjectively considered themselves as monolinguals before entering the study, follow-up questions on language skills showed that only a few (*n* = 7) reported absolutely no knowledge of any other language than Swedish. Although a group of monolinguals could no longer be created in a similar way as in previous studies executed in Umeå, Sweden, it is nonetheless possible that the outcome would have differed in the present study if we would have been able to localize “pure” monolinguals. Therefore, the results of the present study may have limited generalizability across all bilingual groups.

## Concluding Remarks and Further Research

In sum, this study provides no support for proposal that late bilingualism promotes the executive control system in 50–75 year olds. We included both estimated bilingualism and L2 proficiency into the analyses; however, none of these had any influence on inhibitory control or task-switching ability. Results did not differ in sub-analyses of groups that mastered languages with either near or far linguistic distance.

To be able to conclude whether bilingualism can promote cognitive control, future studies would benefit from identifying valid tests of executive functioning that have high correlations and acceptable test-retest reliability. It would also be worth considering tests of cognitive control with ecological validity, as it has been suggested than bilinguals may outperform monolinguals in tests of everyday attention (see Bak et al., 2014a,b; Vega-Mendoza et al., 2015). Since it has been suggested that genetic differences may influence the development of cognitive control in bilinguals (see e.g., Hernandez et al., 2015), future studies should also consider possible interaction effects between genes and language acquisition.

If these goals can be achieved, more studies are needed to explore the long-term term effects of bilingualism on cognitive control. Language learning interventions may also be worth considering since they have recently been suggested to influence cognitive functioning positively in old age (see e.g., Bak et al., 2016).

## Ethics Statement

“Successful aging – A study of how bilingualism and choice of occupation contribute to preserve attention and memory across the adult life span” has been approved by the Regional Ethics Committee at Umeå University. All subjects gave written informed consent in accordance with the Declaration of Helsinki.

## Author Contributions

DS, JL, and PH developed the research questions and wrote the introduction, methods, results, and the conclusion sections. DS performed the formal analyses. All authors have contributed equally.

## Conflict of Interest Statement

The authors declare that the research was conducted in the absence of any commercial or financial relationships that could be construed as a potential conflict of interest.

## Footnotes

1 http://lcad.lab.yorku.ca/
